# EIDM training as a key intervention among researchers to enhance research uptake and policy engagement: an evaluation study

**DOI:** 10.12688/wellcomeopenres.18018.1

**Published:** 2023-02-03

**Authors:** Leila Abdullahi, Hleziwe Hara, Elizabeth Kahurani, Victory Kamthunzi, Lomuthando Nthakomwa, Rose Oronje, Nyovani Madise

**Affiliations:** 1 African Institute for Development Policy, Nairobi, Kenya; 2 African Institute for Development Policy, Lilongwe, Lilongwe, 31024, Malawi

**Keywords:** EIDM training, policy makers, training & mentorship, evidence

## Abstract

In the past decade, the field of Evidence Informed Decision Making (EIDM) has been evolving faster than before. This shows a need for capacity enhancement amongst evidence producers and evidence users in EIDM training. Through the Enhance DELTAS programme, led by the African Institute for Development Policy (AFIDEP), we provided research uptake and policy engagement training, mentorship and webinars to awardees of the Developing Excellence in Leadership, Training and Science (DELTAS) Africa initiative, led by the African Academy of Sciences (AAS).

Two workshops were offered to individual early career DELTAS researchers in policy engagement and evidence uptake, referred to as ENHD101, and among research leaders to enhance institutional capacity on policy engagement and evidence uptake, (ENHD102).

Overall, over the eight months’ period of training, mentorship and webinars, the programme attracted 31 early career researchers and 20 research leaders. Following the programme, the early career researchers understood the importance of EIDM for better health policies and programmes. In addition, the team appreciated the complexities of the policymaking processes as they developed the policy engagement strategy for their research. The utilisation of the EIDM knowledge was reflected during the mentorship with policy briefs as end product.

For research leaders, they appreciated their role in strengthening capacity for EIDM in decision-making spaces. Although during the programme none of the research leaders participated in strengthening capacity for EIDM, the team anticipated improving in the area in the long run. In addition, the research leaders developed and implemented institutional strategies for policy engagement and research uptake through use of social media to influence policymakers.

In conclusion, the project supported capacity building of African researchers in EIDM. It was evident that enhancing knowledge and skills on EIDM through an integrated approach to include training, mentorship, and webinars demonstrated enhanced capacity for policy engagement and evidence uptake.

## Background

Evidence has an important role to play in improving policy, programme and practice decisions that ultimately improve development effectiveness ^1, 2^. Evidence-informed Decision-making (EIDM) is an evolving discipline to help translate the best available evidence into context-appropriate recommendations aligned to the priorities of decision-makers. EIDM is defined as a process where high-quality evidence from research, local data, and professional experiences is synthesised, disseminated, and applied to decision-making in policy and practice ^3– 5^.

The EIDM process is complex as it has to compete with many other factors including interests of policymakers, politics, value systems, individual and institutional capacities, and financial constraints ^6– 8^. Individual and institutional weak capacity for evidence use in policy and programme decisions has attracted a lot of focus in the last decade as one of the many barriers to evidence use ^2, 8, 9^. There are a number of evidence that have investigated the need and efforts to strengthen institutional capacity with the aim of increasing or enhancing the use of evidence in decision-making ^10, 11^. The studies showed a need for a better understanding of efforts to strengthen capacity for evidence use as well as understanding context-specific lessons and insights in building institutional capacity for evidence use.

This paper draws on the Enhance DELTAS programme led by the African Institute for Development Policy (AFIDEP) to strengthen individual capacity for evidence use to include training, mentorship and webinar as a key intervention among researchers and policy makers. Training, mentorship and webinar intervention to enhance research uptake and policy engagement was provided to awardees of the Developing Excellence in Leadership, Training and Science (DELTAS) Africa initiative, led by the African Academy of Sciences (AAS).

The intervention was designed to address the gaps in knowledge and skills for knowledge translation and policy engagement among many DELTAS fellows through the Learning Research Programme (LRP) of the DELTAS initiative ^12^. The Learning Research Programme (LRP) report highlighted institutional weaknesses in promoting knowledge translation and policy engagement practices within DELTAS partner institutions. The institutional weaknesses on evidence use is also mirrored by unpublished PhD research of our DELTAS-funded PhD researcher and AFIDEP staff, who has documented similar weaknesses in her doctoral research (research in progress).

Enhance DELTAS team worked with the first DELTAS Africa programmes to enhance the capacity of individuals, support DELTAS institutions in creating enabling environments for policy engagement and research uptake, facilitate interaction between researchers and policymakers. First DELTAS Africa that the intervention programme targeted is an initiative implemented by the AAS Alliance for Accelerating Excellence in Science in Africa with the support of the Wellcome Trust and the UK’s Foreign Commonwealth and Development Office (FCDO). DELTAS Africa (one) that ended May 2021 was designed to train world class researchers and research leaders in health sciences in Africa and to strengthen the environments in which they operate. The first DELTAS Africa programme supported 11 collaborative teams, spanning 54 lead and partner institutions. DELTAS Africa Phase II that started in 2021 and has introduced a suite of new strategies designed to address gaps identified during DELTAS Africa Phase I. The strategy includes balancing equity and excellence within the constitution of various consortia ^13^. The integrated learning program that included virtual workshops, online self-learning materials including videos, mentorship phase, interactive EIDM at individual and institutional level.

### Research question

1. Can a multi-faceted intervention which combines training and mentoring improve researchers’ knowledge of EIDM and practice?

## Methods

We took a holistic approach at individual level that intends to strengthen individual capacity and existing institutional systems, structures and processes to enable sustained EIDM. The multi-faceted intervention to include training, mentorship and webinar adopted a virtual format. The following integrated approach was used:

a) Consultation with AAS to identify potential trainees: In August 2020, we held virtual consultations with AAS to introduce Enhance DELTAS programme and assess their interest in co-facilitating. The AAS team offered their support to get the programme publicised across the DELTAs family. Through the email circulation from AAS, the interested DELTAS programme expressed interest in our two training modules, ENHD 101 and ENHD 102 which are described fully below.
b) The training intervention components: A tailor-made virtual training targeting early career researchers and research leaders were developed following a needs assessment to understand participants’ needs.
  ○ Training for early career researchers ENHD101 on policy engagement and evidence uptake targeted DELTAS and Africa Early Career Researchers (ECR) undergoing PhD and post-doctoral programme. The key components of ENHD101 were: introduction to principles of EIDM, mapping the health policymaking landscape, developing a policy engagement strategy for research project and knowledge translation and packaging.
  ○ Training for senior researchers and consortium leaders ENHD102, to enhance institutional capacity for policy engagement and evidence uptake was designed for research leaders or senior researchers who were responsible for leading policy engagement and research uptake. Components of ENHD102 were: introduction to EIDM, developing institutional strategies for policy engagement and research uptake, generating demand for evidence uptake and creating an enabling institutional culture for research uptake.
c) Mentorship programme: As part of the learning process, participants were invited to the virtual mentorship programme to help consolidate the learning, build depth and most importantly help them complete their policy products. The mentorship was provided over eight months to monitor their progress on the implementation of their policy engagements tasks. We have used this approach on a number of our programmes with good success rates ^9^. Fellows were assigned a task to complete after the training (for example developing a policy brief based on their research, doing a stakeholder mapping and power-interest matrix, or coming up with a policy engagement strategy).
  Out of the participants trained for ENHD101 and 102, six participants from ENHD101 training for early career researchers (four females and two males) expressed interest in being mentored to develop some evidence products. A reason why some participants chose not to participate was that they were unsure whether they needed the mentorship as yet because they were just starting their research projects. Among those who accepted mentorship, their research ranged from anti-microbial resistance, sexual and reproductive health, strengthening health research and maternal healthcare service utilisation, among others. The fellows were assigned to mentors who supported them up to the end of the programme. During their first meeting, each mentor-mentee pair was asked to complete an agreement outlining the goals and expectations, and a plan for completing at least one evidence product for sharing with relevant policymakers. Out of the six mentees, four mentees developed policy briefs as the evidence product of choice while two dropped out along the way.
d) Webinars: As follow-on support, 2-hour virtual webinar sessions were scheduled in April and May in two key area of interest as expressed by the participants. The webinar topic was derived from the needs assessment. The first webinar conducted in April 2021 was on "How to attain effective context-specific policy engagement strategies" with objective the to familiarise and access policy engagement and evidence uptake toolkits. The second webinar titled “How to effectively use Social Media”, sought to understand why social media is valuable in communicating about research and policy to the public.

### Implementing the EIDM Intervention

Before the training, we conducted a needs assessment and baseline study to understand participants’ capacity development needs. The feedback from this assessment helped us customise the agenda to suit the needs of the participants. From the needs assessment, we identified the following areas of interest: evidence-informed decision-making, context and principles of policy-making, health policies and instruments used by governments, policy engagement and research uptake strategy, evidence synthesis and packaging evidence for non-academic audiences. The first questionnaire was used to gather baseline information on the knowledge about EIDM and policy engagement experience to inform our overall programme evaluation.

### Training sessions

The training on Policy Engagement and Evidence Uptake for Early Career Researchers (ENHD 101) that targeted PhD and post-doctoral early career researchers had 31 participants. The participants came from eight African countries from various DELTAS institution. On the other hand, the training on enhancing institutional capacity for Policy Engagement and Evidence Uptake (ENHD 102) had 21 participants from seven African countries representing various DELTAS programme. The ENHD 102 training targeted research leaders or senior researchers who are responsible for leading policy engagement and research uptake ( Table 1).

**Table 1.** Trainee participants and their DELTAS institutions.

Country	DELTAS Programme affiliation
ENHD 101	
◦ Kenya ◦ South Africa ◦ Nigeria ◦ Namibia ◦ Ethiopia ◦ Tanzania ◦ Ghana ◦ Uganda	◦ KEMRI Wellcome Trust ◦ University of Witwatersrand/ CARTA ◦ Obafemi Awolowo University ◦ Kilimanjaro Christian Medical University College ◦ University of Ibadan ◦ University of Namibia ◦ Addis Ababa University ◦ West Africa Centre for Cell Biology of Infectious Pathogens (WACCBIP), University of Ghana ◦ University of Nairobi ◦ Makerere University ◦ University of Western Cape
ENHD102	
◦ Cameroon ◦ Ghana ◦ Kenya ◦ Mali ◦ Senegal ◦ South Africa ◦ Uganda	1) Developing Excellence in Leadership and Genetic Training for Malaria Elimination in Sub-Saharan Africa (DELGEME) 2) Consortium for Advanced Research Training in Africa+ (CARTA+) 3) Makerere University and UVRI Infection and Immunity (MUII-plus) 4) Sub-Saharan African Consortium for Advanced Biostatistical Training (SSACABT) 5) Initiative to Develop African Research Leaders (IDeAL) 6) Malaria Research Capacity Development in West and Central Africa (MACARD) 7) West African Centre for Cell Biology of Infectious Pathogens (WACCBIP)

1.  ENHD101

This training targeted ECRs who had expressed interest in attending the training (37). We divided them into two cohorts. The first of the ENHD101 training workshop was conducted over five days comprising three virtual hourly slots per day between 26th and 30th October 2020 while the second cohort training was conducted from 25th to 29th January 2021. The first cohort consisted of 19 participants attending the virtual training while the second cohort consisted of 12 participants.

2.  ENHD102

The training workshop for consortium and senior researchers from the DELTAS programme took place between 8th and 11th February, 2021. In total, 22 fellows registered, 20 participated in the full training over three virtual hours daily for four days. The last day of the workshop facilitated a joint workshop between the senior research leaders and policymakers from one of our programmes as well as from West African Health Organisation.

The joint researcher-policymaker interactive workshop was an opportunity to bring together these two groups to discuss ways of enhancing research uptake for decision-making. Four policymakers from West, East, Central and Southern Africa took part in the 3-hour workshop.

### ENHD 101 and 102 training content

Both courses began with a background on the EIDM process as summarised in the content of the training for both ENHD101 and 102 below ( Table 2). The format of the workshop took on a combination of presentations, discussions and group work supported by practical sessions and questions and answers. Further, we invited a Ministry of Health official to share first-hand experience with using evidence within policymaking spaces for the ENHD101 session. Further to this, pre-recorded videos and other self-learning material was prepared to support facilitator-led online learning.

**Table 2.** ENHD 101 and 102 Training Content.

**ENHD 101: Policy Engagement and Evidence Uptake for Early Career** **Number of days**: 4-day 3 hourly sessions **Topics:** 1. Introduction to Principles of Evidence-Informed Decision-Making 2. Mapping the Health Policymaking Landscape. Understanding the policymaking landscapes; understanding the political, social, and economic contexts which influence policymaking; case studies of national, global and regional health policymaking processes. 3. Developing a Policy Engagement Strategy for Research Project. Identifying key stakeholders (stakeholder mapping), stakeholder power interest matrix; effective engagement of policymakers; policy engagement toolkits. “So What?” tools – Embedding monitoring and evaluation and learning in policy engagement and sessions including role play. 4. Knowledge Translation and Packaging. • Rapid synthesis of evidence, translating and packaging evidence in suitable formats for policymakers and non-academic audiences. • Practical sessions - creating research summaries, policy briefs, blogs, and briefing notes.	**ENHD 102: Enhancing Institutional Capacity for** ** Policy Engagement and Evidence Uptake** **Number of days**: 3-day 3 hourly sessions **Topics**: 1. Introduction to Evidence-Informed Decision Making 2. Developing Institutional Strategies for Policy Engagement and Research Uptake – • Engagement objectives, mapping key stakeholders, spheres of influence. • Communications plans- strategic communication tools, collaborating with knowledge translators and the media. • Involving policymakers in research advisory committees; participating in policy advisory committee. • Developing a monitoring and evaluation framework - theory of change, outputs and outcomes to measure. 3. Generating demand for evidence uptake; lobbying for research and knowledge translation funding. 4. Creating an Enabling Institutional Culture for research uptake - EIDM champions, incentives and motivations for research uptake.

**Pre and post course evaluation:** To assess the usefulness of the training, we administered pre-and post-training evaluation questionnaires. The evaluation contained both qualitative and quantitative data that took an average of 15 minutes to complete. The pre-test was offered some minutes before the training was started and the post test was conducted immediately after the training was completed before the attendee dispersed. The pre-and post-test questionnaire were completed online using the Microsoft questionnaire tools. The questionnaire platform was closed 30 minutes after it was dispensed. The pre-course questionnaire consisted of technical questions to understand knowledge of EIDM and to obtain knowledge to inform our training evaluation indicators, with key domain area like EIDM individual capacity through training, mentorship and practice; creation of enabling institutional culture for strategic stakeholder engagements and research uptake; creation of formal and informal interaction between researchers, policymakers and other decision-makers; and how the team contributed to the use of evidence for decision making. The data was collected once, on the first day of training to assess participants’ level of understanding of the technical components. This was administered using the online platform *Survey Monkey*. Immediately after the training the participants were encouraged to complete a post course questionnaire to assess the change in knowledge after the training, and also sought information on the quality of the training, future topics and potential areas of improvement for the training programme. Just as we did for the pre-training evaluation, this was administered at once for all participants using Survey Monkey online platform. The data was based on training materials developed by AFIDEP. For the ENHD101 out of 31 participants who joined the training, 27 (87%) participants completed the pre-post course questionnaire after the training. On the other hand, for the ENHD102, out of 20 participants who attended the training, 8 (40%) participants completed the pre-post survey.

**End line evaluation:** eight months after the project, an end-line evaluation was conducted among the trained team to understand the effectiveness of components or the whole programme achievement of the intended outcomes. The self administered online questionnaire contained both qualitative and quantitative questions that took an average of 15 minutes to complete. However, the questionnaire was sent through an email for the participant to complete at their own convenient time. The endline evaluation questionnaire were completed online using the Microsoft Teams questionnaire tools where the respondents had one month to send back their responses and the survey was closed. Some of the common intended outcomes for both ENHD101 and ENHD102 include developing a policy engagement strategy for their research and developing and implementing institutional strategies for policy engagement and research uptake respectively. For the end-line evaluation, ENHD101 had 15 (48%) respondents while ENHD102 had three (15%) respondents.

The copy of the questions/interview items can be found in the Underlying data ^14, 15^.

## Results

### Pre and post evaluation

The pre-and post-training test was administered and analysed using the survey Monkey software.

### Technical skills developed during the training

***Early career researchers.*** The ENHD101 pre and post course survey results showed that the level of knowledge on EIDM that included various domains as listed in Table 3 before training was 66%, compared to 83% at the end of the training. In addition to the pre-and post-survey assessment, we also evaluated the overall quality of the training. Generally, all the participants rated the quality of the training as very good (30%) and excellent (70%) on a scale of 1 to 5 with 1 being the (lowest) poor, 2 being fair, 3 being good, 4 being very good and 5 being the (highest) excellent. Overall fellow’s understanding of technical aspects improved by the end of the training. For example, knowledge of a well-defined policy question improved by 2.5%, understanding of the streams necessary for the window of opportunity for policy influence increased from 14.7% to 58% and lastly knowledge of the steps in applying evidence synthesis concepts increased from 51% to 87%.

**Table 3.** Pre-post knowledge assessment.

END101	(N=31)		END102	(N=20)	
	Pre	Post		Pre	Post
1. Understand evidence-informed policy:	58	84	1. Understand evidence-informed policy:	57	63
2. Understand process of evidence-informed policy	51	88	2. Understand process of evidence-informed policy	57	75
3. Understand how well- policy question can be defined	97	100	3. Understand how well- policy question can be defined	75	100
4. Understand audiences to communicate research effectively	88	74	4. Understand audiences to communicate research effectively	57	87
5. Understand strategy for bridging the research to policy gap	72	75	5. Understand strategy for bridging the research to policy gap	57	75
6. Understand the window of opportunity for policy influence	15	58	6. Understand the window of opportunity for policy influence	42	62
7. Understand the element of a communications strategy	59	90	7. Understand the element of a communications strategy	57	88
8. Understand the research audiences	56	74	8. Understand the research audiences	57	75
9. Audiences to communicate your research to them more effectively?	70	83	9. Audiences to communicate your research to them more effectively?	71	86
10. Understand steps to applying Evidence synthesis	51	87	10. Understand steps to applying Evidence synthesis	43	71
11. Understand systematic literature review?	73	94	11. Understand how to write for policy influence	43	88
12. Understand narrative literature review?	63	74	12. Understand the differences between conversational writing vs academic writing	57	87
13. Understand how to write for policy influence	76	94			
14. Understand the differences between conversational writing vs academic writing	93	99			
15. Understand things to consider during research presentations for policymakers	73	74			
Average	**66**	**83**	Average	**56**	**80**

***Senior researchers.*** Similarly, the ENHD102 pre and post course survey results showed that the level of knowledge on EIDM that included various domains as listed in Table 3 improved by the end of the training from 56% to 80%. For example, knowledge of the definition of EIDM and stages of the policymaking process improved from 57% to 63% and from 57% to 75%, respectively. Understanding of Kingdon’s three streams necessary for the window of opportunity for policy influence increased by more than double, from about 30% to 62%. Largely, the level of knowledge increased and the participants were generally interested in follow up engagement to support their targeted study areas of interest. The surveys also sought to gauge participants level of satisfaction with the overall design of the training workshop. There was a general consensus in that all participants indicated that the training was effective and it met their expectations. All the respondents rated the quality of the training as “very good” and “excellent”. More results on the training evaluation are included Table 3.

**a)   The training quality**

In addition to the technical knowledge obtained following the training, we also asked participants to assess the training based on things that they liked the most. The following were some of the responses:


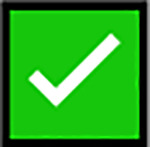
“ *The ease with which the facilitators delivered the training, they are knowledge-packed and interactive which allowed participants to express themselves freely*.”


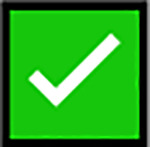
 “ *The content of the presentations and the interactive session were all impactful and engaging. I also like your flexibility in order to achieve the aim of the training*.”


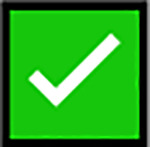
 “ *Learning about what policy is, stakeholder mapping, synthesis of data, writing policy briefs. It has been an amazing course*.”


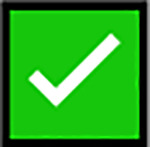
 “ *I like the teaching (presentations). All the presenters are experts in the field and have a lot of knowledge in policymaking*.”


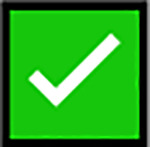
 “ *The discussion on evidence, how strong is the evidence? Reviews and the practical on writing the policy brief among others. The whole programme was wonderful*.”

**b)   Reflections from participants**

We conducted a qualitative survey immediately after the training programme to understand the views of the participants following the training. The participants had the following to say for ENHD101 targeting early career researchers:

*“I am feeling more comfortable to develop a policy brief; I will pay more attention to stakeholder mapping. I will go back to do a paper on systematic reviews which I had initially dropped” (PhD student, ENHD101)*

*“I have seen things from another angle, in pushing my work further regardless of low government interest. I am now more aware of the stakeholders I need to target” (PhD student, ENHD101).*

*“I have learned that it is not always about focusing on publishing but remembering the policy implication. So, for every research, every protocol that I have developed I will be thinking about what is the policy implication for this? How do you want this to end? How many audiences do I want this research to serve? Because if you don’t get it right from the onset then you may find other questions coming up in the end which you didn’t have that data to answer those questions” (Post-doctoral student, ENHD101)*

On the other hand, the ENHD102 participants targeting research and consortium leaders had the following to say:

*“Although exercises are probably a good teaching tool, I think just learning and discussion was good for us now, and having the tools, especially as you have expressed willingness to coach us when we actually need to do these things for real...” (Program manager, ENHD102).*

*“In my experience, access to policymakers and politicians has not been difficult especially where malaria is concerned. Whenever we invite Ministers or we want to discuss an issue with policymakers it is usually easy. However, following these 3 days of workshop, what I am realising is that we have been doing this engagement out of what we have seen other people do or from our gut feeling, but we didn’t have a structured or professional way of approaching it. So there is still a lot of learning on our part to do” (Research leader, ENHD102).*

*“We have worked with researchers for about 5 years. But with COVID it disrupted a lot of things here. I want to say it is possible to improve MNCH with interventions that are evidence-based. We’ve used evidence to move a lot of processes forward for example communication regarding maternal neonatal and child mortality. The communication of evidence has helped to pull a lot of people to trying to see how they can use males in improving access to family planning and child spacing” (Policy makers, ENHD101).*

### Medium-term impact following project end-line survey

Eight months after the training we conducted an end line survey to check on the utilisation of knowledge/skills they obtained during the training. From the respondents, the participants provided positive feedback as to how they have used the skills.

**ENHD101**: The participants responded positively with examples of how they have used the skills.

*Participant 1: I applied to knowledge to write a blog on the potential benefits of my research work (PhD student, ENHD101).*
*Participants 2: I'm developing my research protocol with a view to influencing policy using tips from the training (PhD student, ENHD101).*
*Participants 3: There was a session during the training that covered how to write for different audiences. Used the skill to write a blog article that would be easily understood by a wide audience, both lay and expert (Postdoctoral student, ENHD101).*
*Participants 4: Yes, the training enabled me to write a better literature review chapter for my PhD proposal. It enabled me to think more critically about my literature search. The training also further re-echoed to me that for any grant proposal I am to write, I have think about public health impact of the proposed work and how this will be achieved. The training showed me that for any work, it is important to do stakeholder analysis and take the highlighted stakeholders throughout the research project journey, right from conception of the idea, to implementation and this will make writing policy briefs easier. Thank you so much for the training. I am grateful (PhD student, ENHD101).*

**Figure 1.**
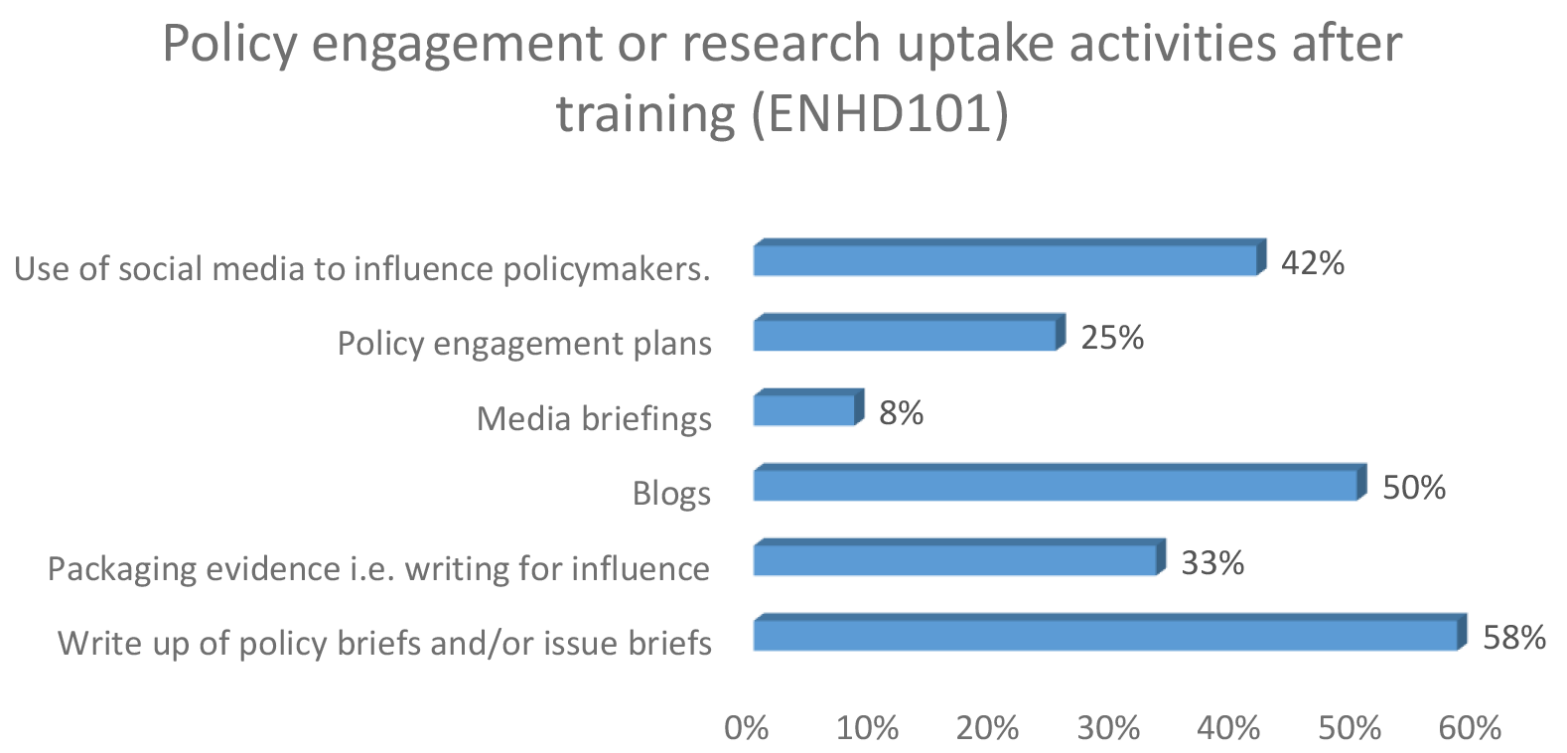
Policy engagement and research uptake following training among early career researchers.

Among the early career researchers, three beneficiaries had written blogs as illustrated in Figure 1 respectively *.*

**ENHD102:** Similar to ENHD101 group, the participants responded positively with examples of how they have used the skills.

*Participant 1: I'm better able to translate and compile important information into smaller snippets to share on our social media pages (Program manager, ENHD102).*
*Participant 2: Yes. I was able to identify the stakeholders in relation to their possible influence on the objective of my policy engagement (advocacy for improved mental health service in Oyo state) (Program manager, ENHD102).*
*Participant 3: I have participated to write a press release to share the result of Sars-Cov 2 ARN sequenced in our Lab in Mali. This brought in the Malian Prime Minister and the Minister of Health to make official visit to our facilities. Also, our Center was contacted before any communication of the government on the evolution of the Covid-19 in Mali (Research leader, ENHD102).*

**Figure 2.**
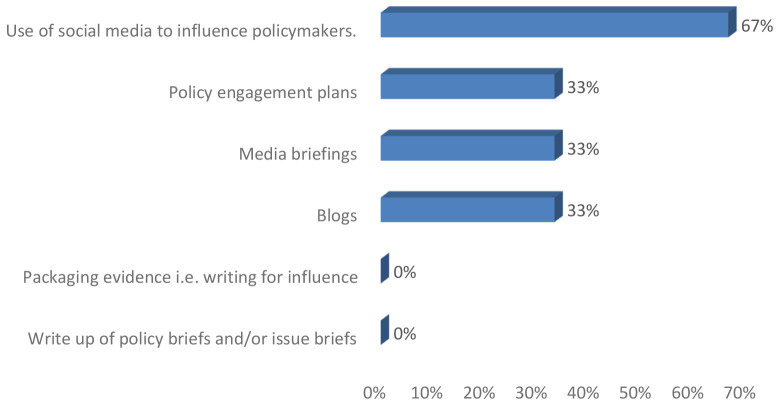
Policy engagement and research uptake following training among senior researchers.

In addition, among senior researchers one beneficiary each reported to have written blog, media brief and policy engagement plans following the training as illustrated in Figure 2. Two senior researchers mentioned that they used social media to influence policy.

### Priority area for future training

**Figure 3.**
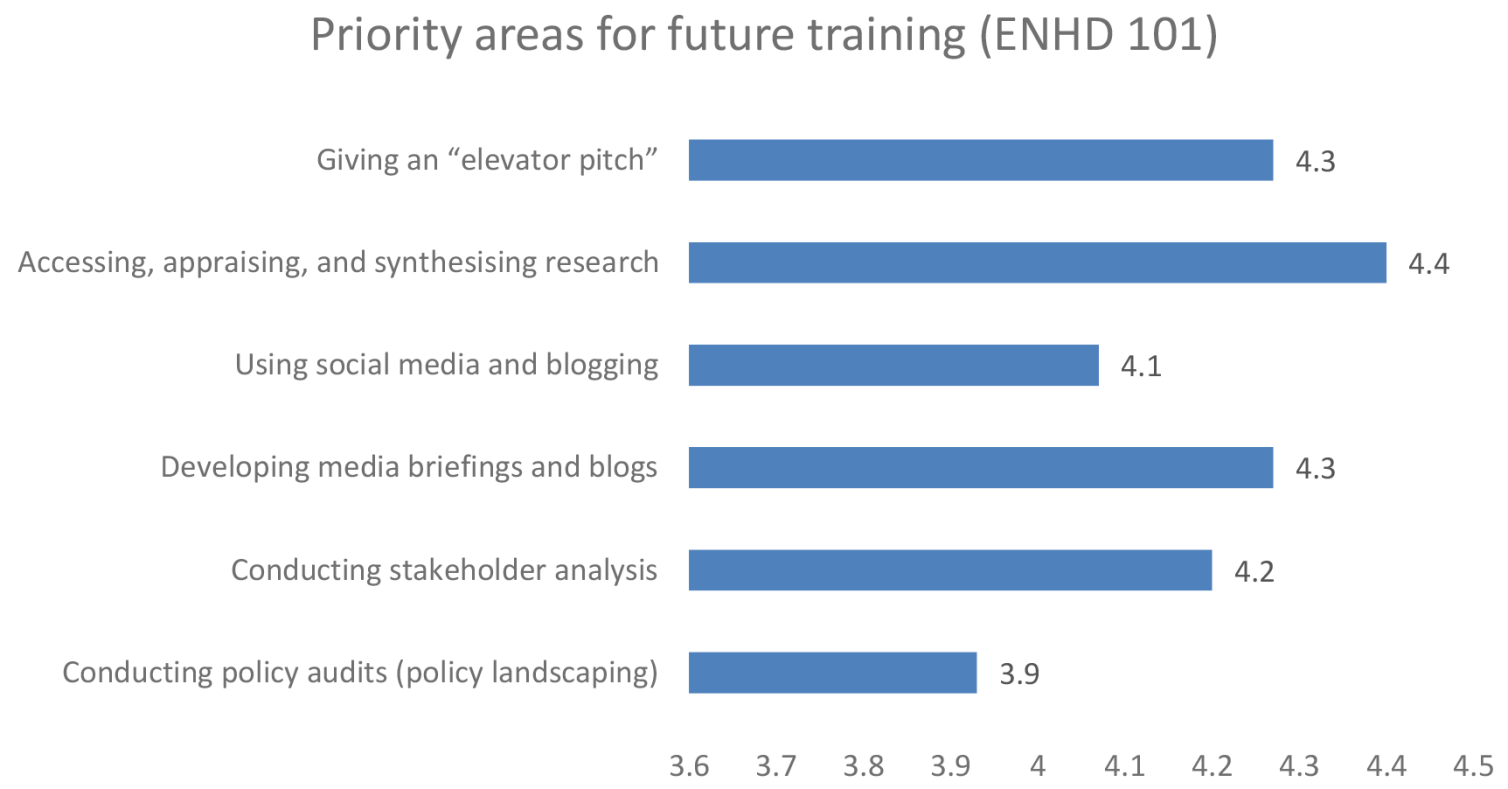
Priority area for future training among early career researchers.

**Figure 4.**
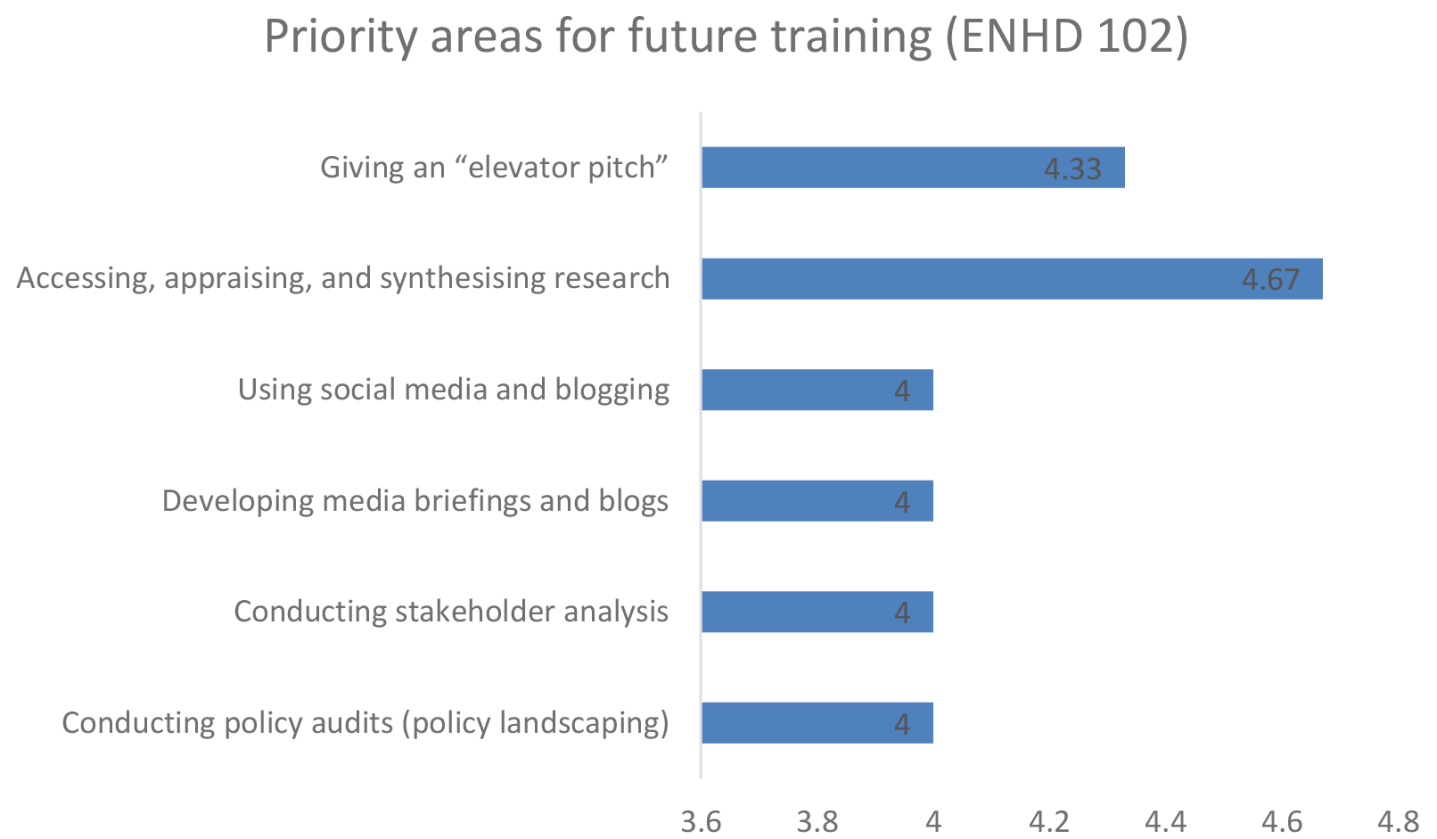
Priority area for future training among senior career researchers.

Further, when participants were asked to recommend on the future training, the early career and senior researchers in unison recommended training on accessing, appraising and synthesising research, developing a media brief and giving an elevator pitch as shown in Figure 3 and Figure 4 respectively.

## Discussion

EIDM has grown interest in the current decade, and so does a need for capacity enhancement both at individual and institutional level amongst evidence producers and evidence users. EIDM is a deliberative process that guides decision-making using best research evidence ^16^. Since 1990s research evidence has traditionally played an integral part in decision-making ^17^. Despite knowledge of EIDM, healthcare organisations around the world have considerable difficulty in translating research evidence into practice ^18, 19^. Barriers to undertaking EIDM include weak capacity for evidence use in policy and programme decisions which includes; a lack of understanding of the value of research evidence; a lack of knowledge of, and engagement in, the process of EIDM; a lack of skill in EIDM; a lack of access to research literature; and a lack of time ^2, 8, 20^.

Evidence has demonstrated capacity development efforts and its implementation to increase or enhance the use of evidence in decision-making. A systematic reviews conducted in 2011 by Clar *et al* had elaborated training among policy makers/influencers as one of the interventions that can harness the use of evidence to inform their decisions. Some of the interventions that had improved the use of research to inform decision making in the study included workshops, tailored messaging for decision-makers, evidence dialogue and capacity building for decision-makers to access and demand for research evidence ^21^. A Hawkes *et al.* 2016 ^8^ documented an experience of capacity building interventions targeting four counties (Bangladesh, Gambia, India and Nigeria) aimed at strengthening capacity for evidence use to inform the decision-making process. The results showed that the interventions were successful in building the capacity of individuals to access, understand and use evidence/data ^8^. However, there are no frameworks to measure the effect of capacity building across various level of policy making cycle.

During the intervention phase, the facilitators and researchers acknowledged the need for leadership skills in engaging stakeholders to enable working better as a team in multi-cultural and multi-sectoral contexts. This is in line with the emerging evidence which shows evidence use in decision-making is enabled by strong leadership and ‘soft’ persuasion skills ^22– 25^. Leadership can use their power to promote and support the EIDM implementation process. Leadership support is considered to be an important facilitator that can act as the champions, initiators, and role models of change interventions to enhance implementation of EIDM culture at both individual and institution level ^26^. Stakeholder engagement was identified within target audience as difficult and yet the most important aspect of EIDM. It is evident that the decision making process is complex with difficulty in getting/engaging researchers and policy-makers that have never worked together to hold dialogue ^22^. However, it is crucial to involve stakeholders from the beginning, and throughout the entire process, to align priorities and foster a common vision towards decision-making and facilitate the uptake of synthesised evidence ^25, 27, 28^.

Following the intervention, it was noted that the demand for skills in how to write for non-academic audiences and policy briefs, and the need to embed in research training was shown through the mentorship phase. In addition, the early career researchers and research leaders agreed that briefs need to be written in clear and jargon-free language. This is because many policy-makers are generalists and do not necessarily come from specific research area. Therefore, as the skills are included in the EIDM process, there is a need to instill writing skills at the start of research, not at the end.

Enhance DELTAS flexibility, in being able to adapt to its target audience requirements, was a key strength of the project. Among the trained group at both early career researchers and research leaders, they identified a critical gap in evidence synthesis and knowledge management capacities, which affected their ability to respond to project objectives. Some of the knowledge gaps that were recommended for future training include accessing, appraising and synthesising research, developing a media brief and giving an elevator pitch. The knowledge gap is anticipated to be addressed in the next phase of this project if it is successfully renewed.

In the study we experience challenges that hindered the intervention to include training, mentorship and webinar intervention with the aim of increasing or enhancing the use of evidence in decision-making. One of the challenges was as a result of the ongoing COVID-19 pandemic, the ENHD 101 training adopted a virtual format as opposed to the originally planned in-person training. This posed challenges in terms of maintaining interactive participation as well as getting the fellows engaged throughout the training. Another challenge experienced was due to poor internet access and connectivity, there was inconsistency in the number of participants who remained online during the sessions. Some of the participants also complained about high internet costs within their countries, hampering them from being fully involved in the training. Time constrain was reported to be a challenge, the feedback from participants indicated that the time to complete exercises was not enough, and more time could be allocated with emphasis to practical sessions. Similarly, some participants were unfamiliar with Microsoft Teams as the training platform, especially in accessing the breakout rooms as well as training material. This resulted in our transferring the training resources to Google Drive for ease of access. Along the way, we shifted to using Zoom platform as most of the participants were familiar with this platform.

Low participation rate was one of the limitations of this evaluation. The drop out of participants was contributed by the virtual modality that seems to be the norm with the global COVID-19 pandemic. 

## Conclusion

Generally, following the intervention, the level of knowledge increased and some of the participants were interested in a follow-up mentorship to support their targeted study areas of interest concerning research uptake and policy engagement. The participants prepared tools and demonstrated various skills for engaging and communicating with policy makers such as blogs and policy briefs. The participants also suggested potential areas that they wish to cover in more details in their future training. Some of these areas include; social media engagement, systematic review and meta-analysis and monitoring and evaluation of the policies. Additionally, it was recommended to consider having such courses integrated within the university curriculum to train the fellows at an earlier stage.

## Ethics approval

The Malawi National Health Sciences Research Committee assessed our study questionnaires as ‘low-risk’ to ethical infringements and waived them from scientific and ethical review in August 2020 (Ref. No. Med /4/36c). The data collection took place between September 2020 to June 2021.

## Consent

The Malawi National Health Sciences Research Committee allowed us to obtain verbal consent (instead of written consent) and use the information of the data, including reporting the study findings anonymously without mentioning the participants’ names.

## Data Availability

Figshare: Combined Pre-post assessment cohort 1& 2 (3).xlsx. https://doi.org/10.6084/m9.figshare.21532461.v2 ^14^ This project contains the following underlying data: ENHD 101 Post-training evaluation Cohort 2-HH.xlsx ENHD 101 Pre-training survey Cohort 2-HH.xlsx (13.35 kB) ENHD 102 Post-training evaluation Knowledge on research uptake -HH.xlsx (13.85 kB) ENHD 102 Pre-training survey -HH.xlsx Figshare: ENHD 102 End-line Assessment.xlsx. https://doi.org/10.6084/m9.figshare.21618252.v3 ^15^ This project contains the following underlying data: ENHD 101 End-line Assessment HH.xlsx (13.66 kB) ENHD 102 End-line Assessment 2-HH.xlsx Data are available under the terms of the Creative Commons Attribution 4.0 International license (CC-BY 4.0).

## References

[1] Stewart R Langer L Erasmus Y : An integrated model for increasing the use of evidence by decision-makers for improved development. *Dev South Afr.* 2019;36(5):616–31. 10.1080/0376835X.2018.1543579

[2] Newman K Fisher C Shaxson L : Stimulating Demand for Research Evidence: What Role for Capacity‐building? *IDS Bulletin.* 2012;43(5):17–24. 10.1111/j.1759-5436.2012.00358.x

[3] Belita E Yost J Squires JE : Measures assessing attributes of evidence-informed decision-making (EIDM) competence among nurses: a systematic review protocol. *Syst Rev.* 2018;7(1):181. 10.1186/s13643-018-0849-8 30390711 PMC6215345

[4] Motani P Van de Walle A Aryeetey R : Lessons learned from Evidence-Informed Decision-Making in Nutrition & Health (EVIDENT) in Africa: A project evaluation. *Health Res Policy Syst.* BioMed Central Ltd,2019;17(1):12. 10.1186/s12961-019-0413-6 30704528 PMC6357392

[5] Yost J Dobbins M Traynor R : Tools to support evidence-informed public health decision making. *BMC Public Health.* 2014;14(1):728. 10.1186/1471-2458-14-728 25034534 PMC4223550

[6] Vogel I Punton M : Building Capacity to Use Research Evidence (BCURE) realist evaluation: stage 2 synthesis report.Hove, United Kingdom: ITAD.2017. Reference Source

[7] Buse K Hawkes S : Health post-2015: evidence and power. *Lancet.* 2014;383(9918):678–9. 10.1016/S0140-6736(13)61945-5 24055453

[8] Hawkes S K Aulakh B Jadeja N : Strengthening capacity to apply health research evidence in policy making: experience from four countries. *Health Policy Plan.* 2016;31(2):161–70. 10.1093/heapol/czv032 25900967 PMC4748127

[9] Oronje RN Murunga VI Zulu EM : Strengthening capacity to use research evidence in health sector policy-making: experience from Kenya and Malawi. *Health Res Policy Syst.* 2019;17(1):101. 10.1186/s12961-019-0511-5 31856848 PMC6923846

[10] Mallidou AA Atherton P Chan L : Core knowledge translation competencies: a scoping review. *BMC Health Serv Res.* 2018;18(1):502. 10.1186/s12913-018-3314-4 29945609 PMC6020388

[11] Tait H Williamson A : A literature review of knowledge translation and partnership research training programs for health researchers. *Health Res Policy Syst.* 2019;17(1):98. 10.1186/s12961-019-0497-z 31842896 PMC6916221

[12] Pulford J Aiyenigba A Liani M : DELTAS Africa Learning Research Programme: Learning Report No. 4 (April 2019–March 2020). 2020.

[13] AAS Open Research: DELTAS Africa – entering a new phase of health research funding.Blog,2019. Reference Source

[14] Hara H Kahurani E Abdullahi L : Combined Pre-post assessment cohort 1& 2 (3).xlsx. figshare. [Dataset].2022. 10.6084/m9.figshare.21532461.v1

[15] Hara H Abdullahi L Madise N : ENHD 102 End-line Assessment.xlsx. figshare. [Dataset].2022. 10.6084/m9.figshare.21618252.v3

[16] Culyer AJ Lomas J : Deliberative processes and evidence-informed decision making in healthcare: do they work and how might we know. *Evidence & Policy: A Journal of Research, Debate and Practice.* 2006;2(3):357–71. 10.1332/174426406778023658

[17] Sackett DL Rosenberg WM Gray JA : Evidence based medicine: what it is and what it isn't. *BMJ.* 1996;312(7023):71–2. 10.1136/bmj.312.7023.71 8555924 PMC2349778

[18] LaRocca R Yost J Dobbins M : The effectiveness of knowledge translation strategies used in public health: a systematic review. *BMC Public Health.* 2012;12(1):751. 10.1186/1471-2458-12-751 22958371 PMC3532315

[19] Schreiber J Stern P : A review of the literature on evidence-based practice in physical therapy. *Internet J Allied Health Sci Pract.* 2005;3(4):9. 10.46743/1540-580X/2005.1089

[20] Ward M Dobbins M Peirson L : Lessons learnt from implementing an organizational strategy for evidence-informed decision-making. *Public health panorama.* 2016;2(03):327–32. Reference Source

[21] Christine C Susan C Lisa D : What are the effects of interventions to improve the uptake of evidence from health research into policy in low and middle-income countries.Final report to DFID,2011. Reference Source

[22] Shroff Z Aulakh B Gilson L : Incorporating research evidence into decision-making processes: researcher and decision-maker perceptions from five low- and middle-income countries. *Health Res Policy Syst.* 2015;13(1):70. 10.1186/s12961-015-0059-y 26621364 PMC4666035

[23] Harries U Elliott H Higgins A : Evidence-based policy-making in the NHS: exploring the interface between research and the commissioning process. *J Public Health Med.* 1999;21(1):29–36. 10.1093/pubmed/21.1.29 10321856

[24] Teng F Mitton C MacKenzie J : Priority setting in the provincial health services authority: survey of key decision makers. *BMC Health Serv Res.* 2007;7(1):84. 10.1186/1472-6963-7-84 17565691 PMC1899487

[25] Turner S D'Lima D Hudson E : Evidence use in decision-making on introducing innovations: a systematic scoping review with stakeholder feedback. *Implement Sci.* 2017;12(1):145. 10.1186/s13012-017-0669-6 29202772 PMC5715650

[26] Peirson L Ciliska D Dobbins M : Building capacity for evidence informed decision making in public health: a case study of organizational change. *BMC Public Health.* 2012;12(1):137. 10.1186/1471-2458-12-137 22348688 PMC3305606

[27] Rycroft-Malone J Seers K Chandler J : The role of evidence, context, and facilitation in an implementation trial: implications for the development of the PARIHS framework. *Implement Sci.* 2013;8(1):28. 10.1186/1748-5908-8-28 23497438 PMC3636004

[28] Ahmad R Kyratsis Y Holmes A : When the user is not the chooser: learning from stakeholder involvement in technology adoption decisions in infection control. *J Hosp Infect.* 2012;81(3):163–8. 10.1016/j.jhin.2012.04.014 22633278

