# Supplementary material for: EIDM training as a key intervention among researchers to enhance research uptake and policy engagement: an evaluation study
Source: Wellcome Open Res. 2023 Dec 8;8:52. Originally published 2023 Feb 3. [Version 2] doi: 10.12688/wellcomeopenres.18018.2 (PMC11074692; doi:10.12688/wellcomeopenres.18018.2)
Supplement: Peer review history

# Reviewer report: Reviewer response for version 2

**DOI:** 10.21956/wellcomeopenres.22431.r113976

**Published:** 2024-12-30

**Authors:** German Andres Alarcon Garavito

**Affiliations:** 1 University College London, London, UK; 2 Universidad de los Andes Facultad de Medicina, Bogotá, Bogotá, Colombia

Thank you for the opportunity to review your work.

 This manuscript provides a detailed description of the Enhance DELTAS programme. It depicts all the steps and methodologies used throughout the experience, such as consultations, training, a mentorship programme, and webinars. Finally, it reports the results following a pre- and post-evaluation structure and includes categories such as new skills, training quality, and, most importantly, reflections from the participants and possible priorities for future programmes.

 However, there are slight points for improvement.

 Results:

 -Could you please add more information regarding the mentorship programme? I perceived an imbalance between this and the other parts of the programme. 

 Discussion:

 -I suggest expanding your reflections by adding more references from similar experiences. I noticed that you referenced a capacity-building intervention in Bangladesh, Gambia, India, and Nigeria.

 It would be good to expand this type of experience so you can contrast your work with theirs. How could experience avoid the limitations of their work and vice-versa?

 -You mentioned the COVID-19 pandemic as a limitation is accurate. I would advise expanding a bit there, what would happen with this programme in a scenario like nowadays?, a non-COVID moment, yet with other threats globally e.g., other pandemics, climate change, wars, etc  

 Overall, this paper is a valuable contribution to the evidence ecosystem. I recommend accepting this paper with minor revisions to address the areas for improvement mentioned above.

Is the work clearly and accurately presented and does it cite the current literature?

Yes

If applicable, is the statistical analysis and its interpretation appropriate?

Yes

Are all the source data underlying the results available to ensure full reproducibility?

Yes

Is the study design appropriate and is the work technically sound?

Yes

Are the conclusions drawn adequately supported by the results?

Yes

Are sufficient details of methods and analysis provided to allow replication by others?

Yes

Reviewer Expertise:

Evidence ecosystems, health systems research, mental health, global health.

I confirm that I have read this submission and believe that I have an appropriate level of expertise to confirm that it is of an acceptable scientific standard, however I have significant reservations, as outlined above.

# Reviewer report: Reviewer response for version 2

**DOI:** 10.21956/wellcomeopenres.22431.r113971

**Published:** 2024-12-17

**Authors:** Maureen Dobbins

**Affiliations:** 1 McMaster University, Hamilton, Canada

This is an important and timely paper and study. Thank you for the opportunity to review it. The authors describe a training and mentoring initiative to improve knowledge and practice among early career researchers and research leaders of African institutions, with the intent of improving EIDM. The overall expectation is that researchers who understand the concepts of EIDM will conduct research that will be used by policymakers because the research will be informed by policymakers.  

 Overall, this is an interesting paper and with some improvements, will make a contribution to the literature.

**General**

 There continue to be grammatical errors throughout the text, including the abstract, which retract from the readability of the paper.

**Methods**

 Please include the needs assessment and baseline questionnaire as an appendix. Was an reliability and validity of these measures conducted. Were there tools developed from the literature? Or created new? If based on pre-existing tools, these should be cited.

 Please add another sentence of two providing more specifics about how recruitment for both 101 and 102 were conducted.

 For 102, in paragraph above table 1 you say 21 leaders participated, but in the paragraph below the table you say 20 participated. Please clarify.

 In the description of 102, I think it could all be one paragraph. How were policymakers identified to participate in the 3 hour workshop on the last day with research leaders.

 Pre-Post questionnaire

 Did 101 and 102 participants complete the same pre-post questionnaire. It seems like it would have been two separate questionnaires, given the content of the two trainings differed, but this is unclear.

 It would strengthen the paper to include the full questionnaires in the appendix, and examples of specific questions included in the text of the methods section. How was the questionnaire developed? Was it informed by previously existing tools? If yes, it should be described how they were used and they should be cited. If previously existing tools were not used, rationale for why not should be articulate. Was the questionnaire tested for reliability and validity ahead of time? Why not, if not done.

 All the same questions as above apply for the end-line evaluation. Did you send reminders to participants to complete the questionnaire over the month?

 The statement the questions can be found in the underlying /extended data should be moved to near the beginning of the description of this section on the evaluation measures. This way the reader learns up front they can go look at the full questions if they want to know more specifics about the questions. However, while it is wonderful to have the data included, the actual questions are also needed.

**Results**

 For the pre-post evaluation how was this assessment done specifically. For example, was the change for each individual per question noted, and the change scores for each participant assessed to determine overall change from pre to post. Or was an average (from 1-5) for each question calculated and compared pre-post. These details on the analysis should be included in the Methods section.

 From Table 3, it appears to me that the questions may assess participants perceptions of the concepts rather than assess their actual knowledge of the concepts. It is difficult to determine what was actually assessed without including the questions as they actually appeared.

 For the qualitative data collected post training (immediate and 8 months post), please include in the Methods what qualitative analysis methods were used to analyze this data.

 For the priority areas for future training, please include in the Methods a bit more about how this data was collected and analyzed. Looks like you used a 5 point scale. Did participants right out these statements, or did you identify them and have participants rate their importance for future training.

**Discussion**

 The discussion could be further enhanced and compared and contrasted to the literature. Please expand on implications for the future for this training; and how to improve participation and completion rates. Also please say a bit more about how future studies can objectively measure is attainment of the knowledge and skills through this program by early career researchers and research leaders results in uptake of research by policymakers.

Is the work clearly and accurately presented and does it cite the current literature?

Partly

If applicable, is the statistical analysis and its interpretation appropriate?

Partly

Are all the source data underlying the results available to ensure full reproducibility?

Yes

Is the study design appropriate and is the work technically sound?

Partly

Are the conclusions drawn adequately supported by the results?

Yes

Are sufficient details of methods and analysis provided to allow replication by others?

Partly

Reviewer Expertise:

EIDM, knowledge translation, capacity development, public health

I confirm that I have read this submission and believe that I have an appropriate level of expertise to confirm that it is of an acceptable scientific standard, however I have significant reservations, as outlined above.

# Reviewer report: Reviewer response for version 2

**DOI:** 10.21956/wellcomeopenres.22431.r78522

**Published:** 2024-05-06

**Authors:** Bey-Marrie Schmidt

**Affiliations:** 1 University of the Western Cape, Cape Town, South Africa

This article describes the results for a multi-faceted intervention that combines training and mentoring to improve researchers’ knowledge of EIDM and practice? This study consists of consultations with trainees, the implementation and evaluation of training and mentorship aspects, and follow-up webinars. The various aspects are well-described, a pre- and post-questionnaire was administered right before and after the training, and an end-line evaluation was conducted. The results are detailed and provide evidence of participants' experiences. The discussion is appropriate and the authors detail gaps in research and practice, however, it would have been more beneficial if they had suggested potential ways of addressing these gaps. For example the authors state "However, it is crucial to involve stakeholders from the beginning, and throughout the entire process, to align priorities and foster a common vision toward decision-making and facilitate the uptake of synthesized evidence". They could have perhaps noted the role of integrated knowledge translation in this type of study. Overall, it is an interesting article and offers valuable findings.

Is the work clearly and accurately presented and does it cite the current literature?

Yes

If applicable, is the statistical analysis and its interpretation appropriate?

Yes

Are all the source data underlying the results available to ensure full reproducibility?

Yes

Is the study design appropriate and is the work technically sound?

Yes

Are the conclusions drawn adequately supported by the results?

Yes

Are sufficient details of methods and analysis provided to allow replication by others?

Yes

Reviewer Expertise:

Evidence-informed decision-making, evidence synthesis, qualitative research, knowledge translation, community engagement.

I confirm that I have read this submission and believe that I have an appropriate level of expertise to confirm that it is of an acceptable scientific standard.

# Reviewer report: Reviewer response for version 2

**DOI:** 10.21956/wellcomeopenres.22431.r74318

**Published:** 2024-04-08

**Authors:** Jana Groß Ophoff

**Affiliations:** 1 Institute for Secondary Education, University College of Teacher Education Vorarlberg, Feldkirch, Austria

Dear authors, dear editorial board,

 Thank you very much for the opportunity to review the article “EIDM training as a key intervention among researchers to enhance research uptake and policy engagement: an evaluation study”, which describes the effects of a training program for young and senior researchers in Africa to improve their Evidence Informed Decision Making.I recommend a major revision.

 The major part of the article consists of the description of the organization and implementation of the training. It contains a lot of **redundancies** and needs to be streamlined. The mass of **abbreviations** complicates the comprehensibility – why is it important for readers to know (and remember) all of them? This is not at all necessary and I strongly recommend the authors to check to what extent these are needed. For example, why is the abbreviation ENHD101 used to identify the first intervention? For readers to understand, that there were two different trainings, the terms “intervention 1” and “intervention 2” or the like would be sufficient.

 Overall, the article requires a **clearer structure**. In particular, the description of the training and the context in which they took place belong in the methods section of the article, but make up a large part of the theoretical section of the paper. This section in turn only touches briefly, but accurately EIDM, but as this is at the heart of this work, it should be given more space. This is a well-established topic of research in different disciplines. An example for an interdisciplinary network dealing with this topic can be found here: https://transforming-evidence.org/projects/transforming-evidence-network. What would be way more interesting for international readers (and is missing in the introduction/theory section), is an explanation of the African context and the health sector, and why is EIDM important at all. This can also provide the framework for a much more elaborate discussion of the results (see below).

 A lot of the **decisions** made are not transparent and must be better substantiated. For example, I wasn't sure which stakeholders took part in the training (and why) until the end of the paper: Researchers, policy makers, or who? One reason for that was the repeatedly mentioned objective of supporting “research uptake and policy engagement” of “evidence producers and evidence users”, but isn’t the former part of being a researcher anyway and why are the participants evidence users? Other aspects, that are not sufficiently substantiated are, why young and senior researchers were invited to participate in two separate trainings, why the two different trainings are investigated in the same paper, but not at all compared? Accordingly, Fig.1/2 and Fig. 3/4 should be considered to be merged (and statistical analysis carried out). Even though the research design is described in the methods section, it needs to be straightened (redundancies). The difference between the pre- and post-course evaluation and the end-line evaluation is not easy to follow. One reason for this is that the actual survey is not described in detail: What did the survey actually assess? Were there parallel versions available? And why are there differences in the platforms used to carry out the surveys? And what do the authors mean by technical skills or knowledge? Do they mean research capabilities or competencies (which are necessary for well-reflected decision-making) or really technical skills? From what is reported in Table 3, one comes to the conclusion that self-ratings about the ability to carry out certain EIDM steps were assessed, that are not at all an operationalization of skills or knowledge (for which it can be identified, what the correct solution is). This means that a more profound description of the used research instruments is necessary, and if the authors assessed in fact self-ratings, they need to be more precise in their terminology. For example (if I am right), they investigated the self-rated ability to make evidence-informed decisions as an outcome of a training… here, another theoretical aspect is missing in the theoretical section of this article: Which form of learning and learning gains are to be expected of such a training? For example, self-ratings of abilities are closely related to concepts of self-efficacy beliefs (Bandura, 1997) that are (as an indicator of motivation) reportedly of relevance to EIDM. Connected to that, the research question should be revised, too.

 The **results** section contains a lot of interesting results, particularly the quotes on page 8. However, these need to be framed accordingly, both in the introductory part and in the discussion of the paper (see above). In its current form, the article remains at the level of an evaluation report and as mentioned, does not fully develop the potential to provide insights into how training for EIDM can motivate and impart strategies. And as a researcher in the field of EIDM (even though in another discipline), this is the truly interesting part of this paper, as the research field in general struggles to develop trainings that are able to convince and empower stakeholders to carry out EIDM. If possible, it would be interesting, too, to learn not only about the content of the training that was liked most (which is not associated with knowledge gains, but with the motivation to get involved), but what was missing. In principle, this is echoed in “3. Priority area for future training”, but that is not enough, especially as contradictions arise here that need to be addressed. Why, for example, do emerging and senior researcher recommend “training on accessing, appraising and synthesising research”? Haven’t they been already educated in that regard – or how did they become researchers? For example, why did one participant report “Yes, the training enabled me to write a better literature review chapter for my PhD proposal” and how was this connected to the objective of the training? At this point at the latest, the suspicion arises that the training was more like a tutoring session for researchers - or was the aspiration more than that? Another contradiction not addressed is the result, that senior researchers rated their EIDM knowledge (pre-test) lower compared to the emerging researchers (Table 3). Is this the typical effect that experts are better in appraising their abilities than novices (for which is a lot of evidence available, too) or is it an effect of the education system (for example reforms of study programs)?

Is the work clearly and accurately presented and does it cite the current literature?

No

If applicable, is the statistical analysis and its interpretation appropriate?

No

Are all the source data underlying the results available to ensure full reproducibility?

No source data required

Is the study design appropriate and is the work technically sound?

Partly

Are the conclusions drawn adequately supported by the results?

Partly

Are sufficient details of methods and analysis provided to allow replication by others?

Partly

Reviewer Expertise:

Data-based decision-making (Education), Evidence-Informed decision-making in higher and continuous education.

I confirm that I have read this submission and believe that I have an appropriate level of expertise to state that I do not consider it to be of an acceptable scientific standard, for reasons outlined above.

## References

[1] : Self-efficacy. 2000; 10.1037/10522-094 212-213 10.1037/10522-094

# Reviewer report: Reviewer response for version 1

**DOI:** 10.21956/wellcomeopenres.19978.r58252

**Published:** 2023-06-08

**Authors:** Kristen Lwin

**Affiliations:** 1 School of Social Work, University of Windsor, Windsor, Ontario, Canada

I’m not sure what “There are a number of evidence that have investigated the need and…” means.

 Page 4 - Details about the intervention are required – how many hours of training, how many hours and meetings of mentorship, and number of webinars, etc.

 Page 5 - Details about ENHD 101 – It’s not clear what “37” in the first sentence means. If this is the sample size include it in parentheses.

 Methods section:

- It is not clear what the pre- and post-test scores mean or are scored out of - details for all measures must be provided. There is currently almost no methods details.

Results section:

- Quality of training – “generally, all the participants…” must be explained in greater details. Provide frequencies for results.
- Were there quantitative results for the 8 month follow up? I believe the same survey (as the pre- and first post-tests) were administered.

Discussion section:

- Results should be integrated into the wider body of literature.

Overall - Edit for grammar, use of consistent verb tense, flow, and concision throughout the manuscript.

Is the work clearly and accurately presented and does it cite the current literature?

Partly

If applicable, is the statistical analysis and its interpretation appropriate?

Partly

Are all the source data underlying the results available to ensure full reproducibility?

No

Is the study design appropriate and is the work technically sound?

Partly

Are the conclusions drawn adequately supported by the results?

No

Are sufficient details of methods and analysis provided to allow replication by others?

No

Reviewer Expertise:

Quantitative Methodology, Statistical Analysis, Program Evaluation

I confirm that I have read this submission and believe that I have an appropriate level of expertise to confirm that it is of an acceptable scientific standard, however I have significant reservations, as outlined above.

# Reviewer report: Reviewer response for version 1

**DOI:** 10.21956/wellcomeopenres.19978.r54701

**Published:** 2023-03-10

**Authors:** Kirchuffs Atengble

**Affiliations:** 1 PACKS Africa, Accra, Ghana

Firstly, the entire article needs to be revised to improve its ease of reading, as grammatical constructs (and obscure connections in sentences) makes current reading a bit difficult. Consistency and coherence in idea communication should critically be looked at; e.g. "Through the email circulation from AAS, the interested DELTAS programme expressed interest in our two training modules, ENHD 101 and ENHD 102 which are described fully below". Do the authors mean the programme itself, or interested programme partners showed interest?

 Quite unconventionally also, the article was written in active voice, using first person plural pronouns. This is however left to the discretion of journal editors.

 The methods described suggests that different kinds of evaluations (needs assessment, pre-post, endline) were being reported. These should be clearly differentiated in the findings/Results section, and better articulated.

 Quality of the (learning/intervention) evaluations could have been improved if demographic data on learners were used to evaluate their respective degree of learning, producing insights to support learning among particular demographics.

 It is unclear if the discussion portion of this article was truly dedicated to findings of the study, or random review of the literature. These should have a common focus, linked to the different findings/results reported.

 The conclusion will need to be adequately revised to coalesce lessons from the different findings from the evaluation. Potentially, the authors could explore implications for program management, intervention design, among others. This is an evaluation.

Is the work clearly and accurately presented and does it cite the current literature?

Yes

If applicable, is the statistical analysis and its interpretation appropriate?

Not applicable

Are all the source data underlying the results available to ensure full reproducibility?

Partly

Is the study design appropriate and is the work technically sound?

Partly

Are the conclusions drawn adequately supported by the results?

Partly

Are sufficient details of methods and analysis provided to allow replication by others?

Partly

Reviewer Expertise:

Research methods, EIDM, knowledge management, public policy, decision making, international development, information systems, organisation development

I confirm that I have read this submission and believe that I have an appropriate level of expertise to confirm that it is of an acceptable scientific standard, however I have significant reservations, as outlined above.
